# Laparoscopic iliopubic tract repair to treat recurrent pediatric inguinal hernia

**DOI:** 10.1007/s00464-021-08776-5

**Published:** 2021-10-25

**Authors:** Sung Ryul Lee

**Affiliations:** Department of Surgery, Damsoyu Hospital, 234 Hakdong-ro, Gangnam-gu, Seoul, Republic of Korea

**Keywords:** Pediatric inguinal hernia, Laparoscopy, Re-operation, Iliopubic tract, Re-recurrence

## Abstract

**Background:**

Congenital defects, such as open processus vaginalis and the canal of Nuck, are common causes of primary pediatric inguinal hernia (PIH). However, in some patients, PIH occurs via acquired defects rather than congenital defects. The most representative cause of PIH is recurrent hernia. Recurrent PIH is treated with high ligation (HL), which is the same method that is used to treat primary PIH. However, the re-recurrence rate of recurrent PIH is high. This study aimed to compare laparoscopic iliopubic tract repair (IPTR) with laparoscopic HL for the treatment of recurrent PIH after primary PIH repair.

**Methods:**

From June 2013 to March 2019, 126 patients (< 10 years old) with recurrent PIH were retrospectively enrolled. Patients were divided into two groups according to the operative technique: laparoscopic HL (58 patients) and laparoscopic IPTR (68 patients). With HL, the hernial sac was removed and the peritoneum closed. With IPTR, iliopubic tract and transversalis fascia sutures were applied.

**Results:**

There were no cases of conversion to open surgery. Re-recurrence only occurred in the HL group; no patients in the IPTR group developed re-recurrence (8.6% [5/58] vs. 0.0% [0/68], respectively; *p* = 0.044). The mean duration from re-operation to re-recurrence in these five patients was 10.6 months. Other surgical outcomes and complications did not differ between the two groups.

**Conclusions:**

Laparoscopic IPTR is an effective surgical treatment for reducing re-recurrence of recurrent PIH.

Pediatric inguinal hernia (PIH) is a common disease in children. The incidence of PIH is approximately 3–5% in term infants and 9–11% in preterm infants [1]. Congenital defects, including open processus vaginalis (PV) in males and open canal of Nuck (CN) in females, account for most cases of primary PIH [1]. Moreover, PIH can be acquired in some patients, such as from recurrent hernia. Because PV and CN are closed in the first operation, recurrent PIH is classed as an acquired cause of PIH. The main treatment for primary PIH and recurrent PIH is high ligation (HL). There are few reports on the re-recurrence rate after re-operation for recurrent PIH, but re-recurrence rate has been reported high [2]. Treatment of primary PIH due to congenital defects and recurrent PIH due to acquired defects involves different surgical methods.

PIH is usually treated in two ways: open repair or laparoscopic repair with HL. The recurrence rate after laparoscopic repair is 1.1–18.6% [3, 4]. There have been many reports of HL as a treatment for recurrent PIH [5, 6]. In addition, there have been many reports of tissue reinforcement of the internal ring as a treatment for recurrent PIH [7–9]. The re-recurrence rate of recurrent PIH ranges from 0.0 to 4.2% in previous studies, although the surgical method and follow-up period differed between studies [2, 5–8]. For treatment of adult recurrent inguinal hernia, large mesh implantation is recommended to reduce re-recurrence, which is distinct from primary inguinal hernia treatment [10], while prosthetic mesh should never be considered for recurrent PIH repair [1].

Laparoscopic repair is performed in the context of primary and recurrent PIH treatment. Two widely performed laparoscopic methods are used to treat recurrent PIH: laparoscopic transabdominal closure (LTAC) and laparoscopic percutaneous extraperitoneal closure (LPEC) [5–8]. There are differences between these methods in terms of presence or absence of hernial sac removal, use of absorbable versus non-absorbable ties, HL, and tissue reinforcement.

Despite several options being available, no general guidelines have been established for the treatment of recurrent PIH. Iliopubic tract repair (IPTR) is a tissue reinforcement method used for laparoscopic PIH treatment [11, 12]. The iliopubic tract was first described by Alexander Thomson [13] and is identified as a thickening of the transversalis fascia running deep and parallel to the inguinal ligament [14]. Several reports have described the results of laparoscopic IPTR for the treatment of recurrent PIH [7, 9].

One previous report classified primary PIH and tailored surgery accordingly; however, this was a case of open surgical repair, not laparoscopic repair [15]. Unlike in adults, technical details affect recurrence rates in pediatric patients, because synthetic mesh is not used in PIH repair. The author hypothesized that IPTR for the treatment of recurrent PIH would reduce the rate of re-recurrence compared with HL, which is usual for primary PIH treatment. No studies have compared HL with IPTR for the treatment of recurrent PIH. Thus, this study aimed to compare IPTR with HL for the laparoscopic treatment of recurrent PIH.

## Materials and methods

All procedures performed in studies involving human participants were in accordance with the ethical standards of our institutional and/or national research committee and with the 1964 Helsinki Declaration and its later amendments or comparable ethical standards. This study was approved by the Institutional Review Board of Damsoyu Hospital (DSY-2021-001). Informed consent was obtained from patients’ parents/guardians.

**Fig. 1:**
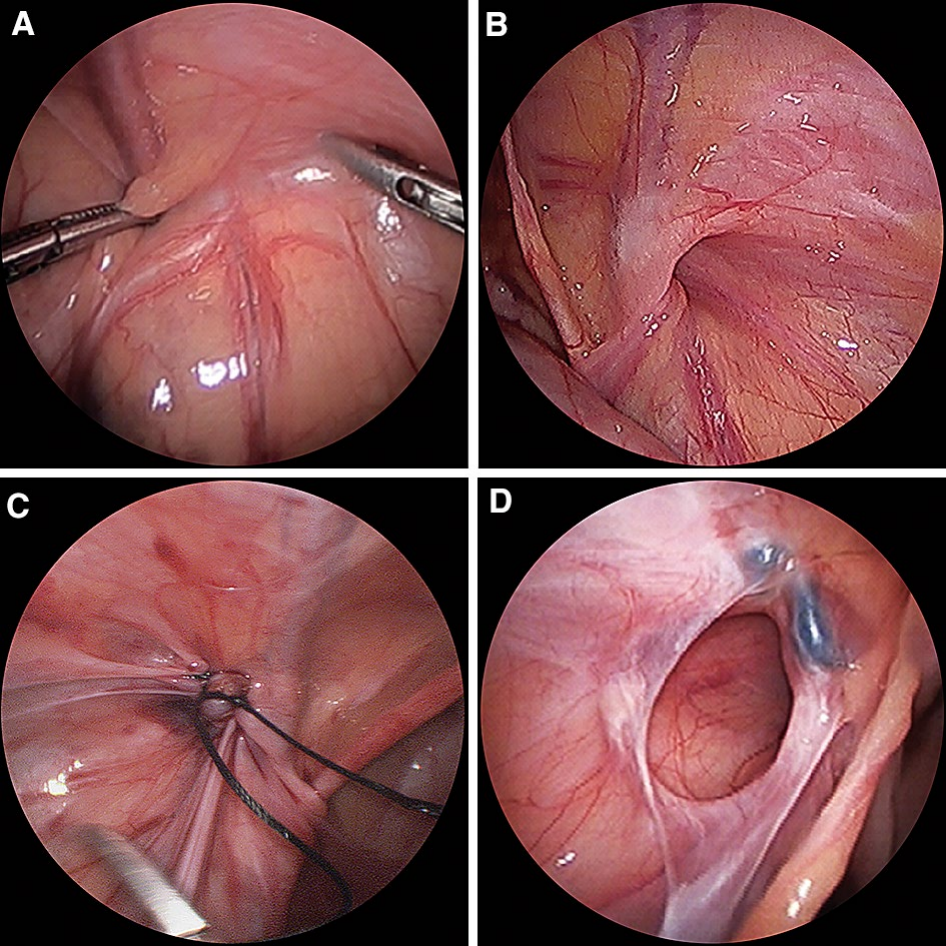
Acquired PIH as recurrence and MCIH. **A** Closure of processus vaginalis during laparoscopic exploration 2 years ago. **B** MCIH developed 2 years later. **C** Laparoscopic repair of left inguinal hernia 11 months ago. **D** Recurrent PIH occurred 11 months later. *PIH* pediatric inguinal hernia; *MCIH* metachronous contralateral inguinal hernia

There are some cases on acquired PIH. The first example is recurrent PIH (Fig. 1A), which occurred after ligation of a congenital defect during primary repair. The second example is of a metachronous contralateral inguinal hernia (MCIH) (Fig. 1B), which occurred after identifying closed PV or CN during primary repair [16]. In our institution, laparoscopic repair is performed for both recurrent and primary PIH. In the present study, all patients underwent laparoscopic repair and there was no conversion to open repair.

### Inclusion and exclusion criteria

**Fig. 2:**
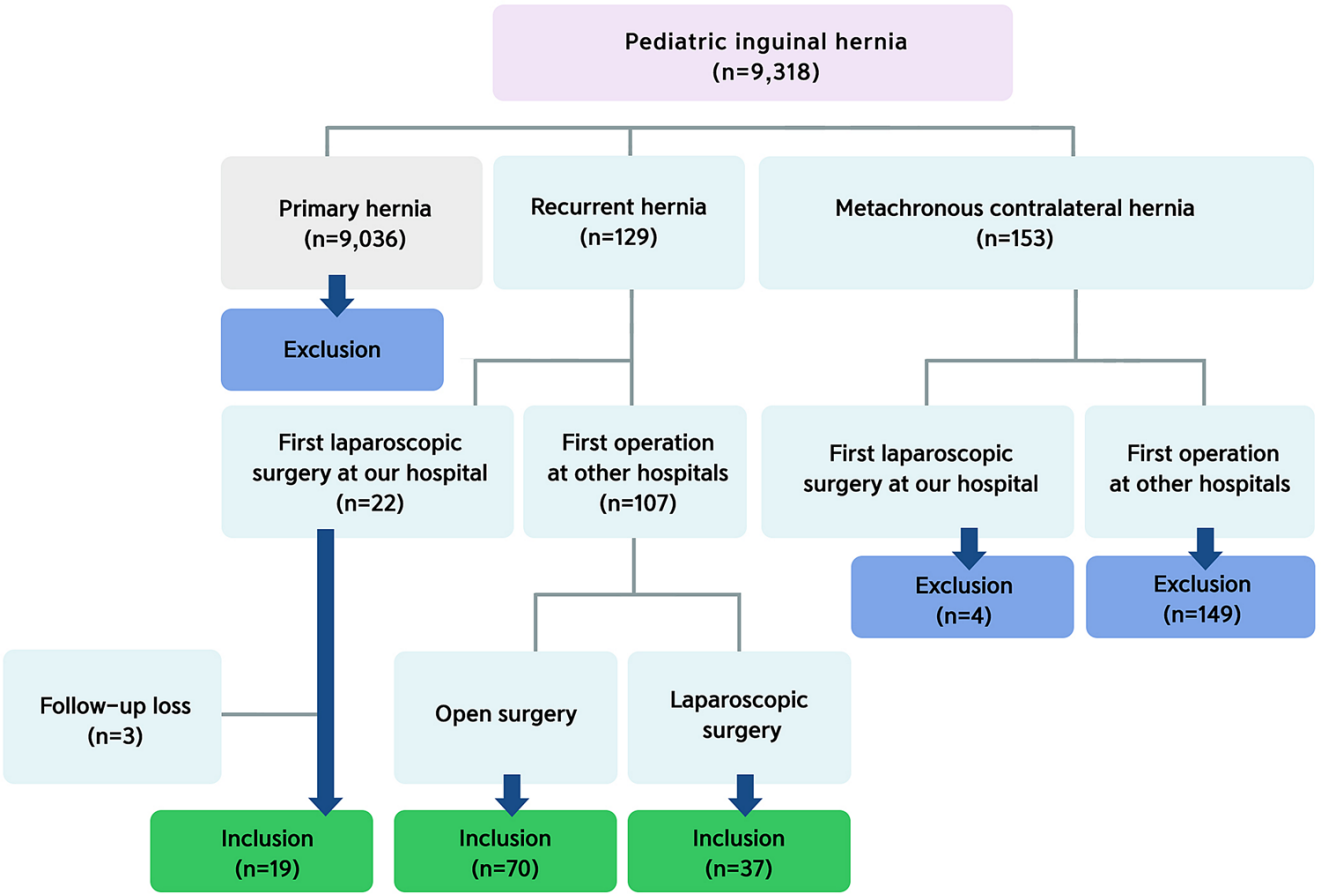
Selection of patients with recurrent PIH. *PIH* pediatric inguinal hernia

This study retrospectively analyzed case records of 126 patients who presented with recurrent PIH from June 2013 to March 2019 at Damsoyu Hospital, Seoul, Republic of Korea. During the study period, 9,318 patients underwent laparoscopic repair for PIH. Of these patients, 9,036 had primary PIH. A total of 153 patients with MCIH were excluded from the study. The inclusion criteria included re-operation at our hospital, regardless of whether the primary operation was performed at our hospital or not. The exclusion criteria included re-operation at an outside hospital. Three patients with recurrent PIH underwent primary repair at our hospital, but they were excluded from this study because of follow-up loss. A total of 126 patients were finally analyzed (Fig. 2), including patients who underwent open or laparoscopic primary repair, either at our hospital (*N* = 19) or at other hospitals (*N* = 107).

### Group classification

From June 2013 to September 2016, children underwent laparoscopic HL for the treatment of recurrent PIH. Re-recurrence was observed in two patients until September 2016; the treatment method was then changed to laparoscopic IPTR for recurrent PIH beginning in October 2016. Each patient’s history and surgical method were confirmed by checking the medical records obtained from the facility at which the initial procedure had been performed. All data were retrospectively collected by a data manager at our research center. After the removal of the hernia sac, the hernia defect size was measured using the length of the laparoscopic instrument tip. We compared demographic and surgical outcomes between the IPTR and HL groups. We analyzed the primary operative method, hernial laterality, protruded organs, duration from first operation to re-operation, operation time, length of hospital stay, complications, and re-recurrence rate.

### Laparoscopic IPTR technique

**Fig. 3:**
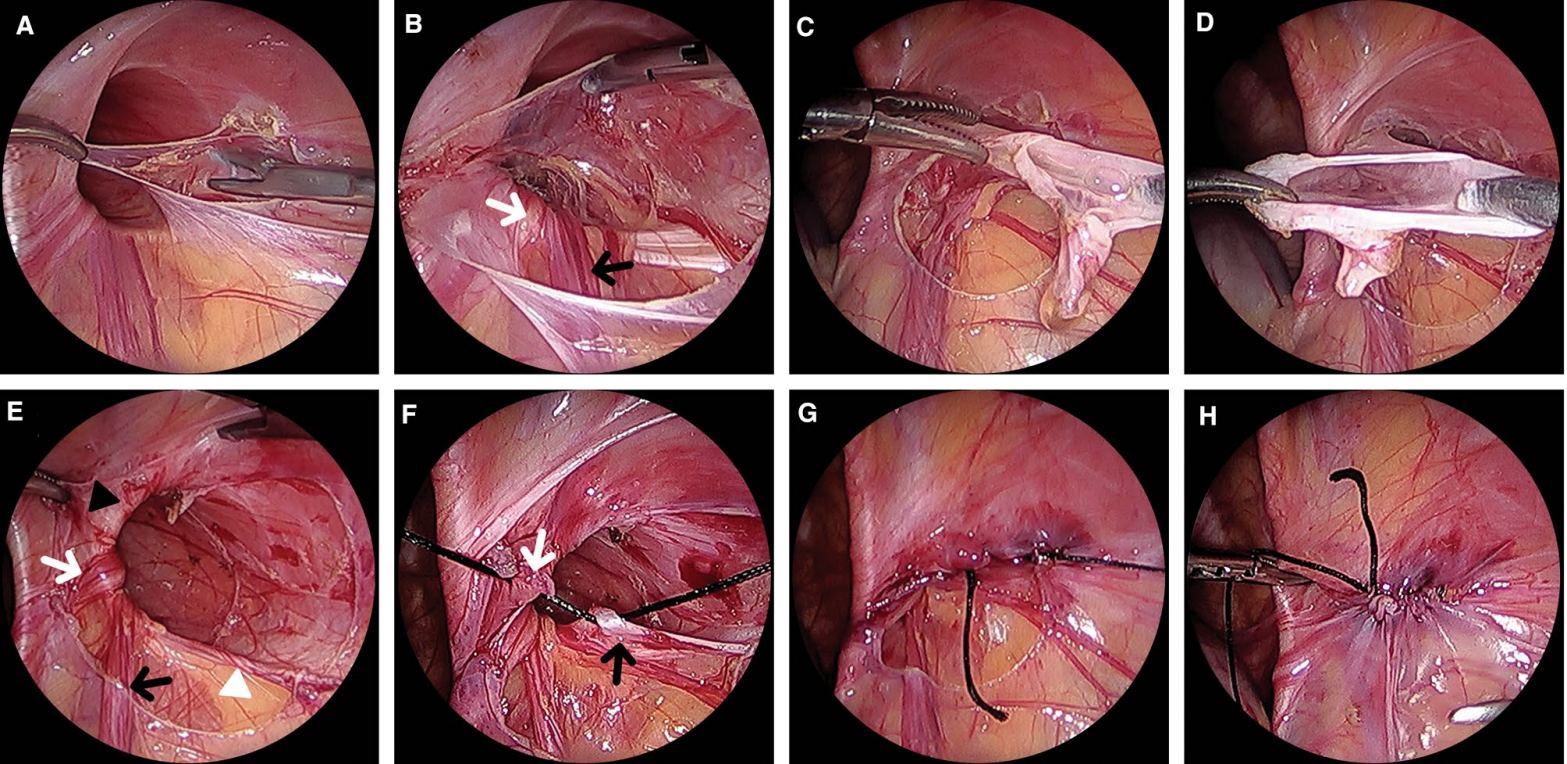
IPTR procedure. **A** The hernial sac was incised at the lateral side of the internal inguinal ring. **B** The vas deferens (white arrow) and spermatic cord (black arrow) were separated from the hernial sac. **C** The entire hernial sac was transected. **D** The hernial sac was completely removed. **E** Internal inguinal ring: the iliopubic tract (white arrowhead) and the medial muscular arch of the transversalis abdominis (black arrowhead). Preservation of the vas deferens (white arrow) and spermatic cord (black arrow). **F** First stitch of iliopubic tract repair. Iliopubic tract (black arrow) and medial muscular arch of the transversalis fascia suture (white arrow). **G** Completion of IPTR. **H** Complete peritoneal repair. *IPTR* iliopubic tract repair

All procedures were performed under general anesthesia with patients in the supine position. Laparoscopic repair was performed using a three-port technique. The laparoscopy system included a 3.0-mm camera and 3.0-mm instruments. A transumbilical 3.0-mm incision was made, and a 3.0-mm trocar was used to create carbon dioxide pneumoperitoneum that was maintained at 6–8 mmHg. Two other 3.0-mm instruments were inserted through separate 3.0-mm stab incisions bilaterally on the abdomen. With HL surgery, the hernial sac was removed and the peritoneum closed. The whole process of laparoscopic IPTR is shown in Fig. 3. The hernial sac was incised at the lateral site of the entrance to the internal inguinal ring. After separating the vas deferens and spermatic cord from the hernial sac by gentle retraction, the entire hernial sac was removed. In female patients, the round ligament was separated from the hernial sac and preserved. Dissecting the hernial sac revealed the anatomy of the hernia. Suture was performed when the iliopubic tract on the inferolateral side of the internal inguinal ring and the transversalis abdominis muscle arch on the superomedial side were identified. The iliopubic tract was sutured using non-absorbable 3–0 silk suture, as previously reported [11].

### Statistical methods

All statistical analyses were performed using R software version 3.6.1 (R Development Core Team, Vienna, Austria; http://www.R-project.org). Continuous variables are presented as mean and range, whereas categorical variables are presented as frequency and percentage. The Wilcoxon rank-sum test was used to test for normality of continuous variables. Fisher’s exact test or the *χ*^2^ test was used for categorical variables. A *p* value threshold of 0.05 was chosen, and any univariate *p* value of ≤ 0.05 was considered statistically significant. Finally, odds ratios were calculated using Fisher’s exact test and logistic regression.

## Results

Patients’ characteristics are shown in Table 1. All patients with recurrent PIH had indirect hernia. There were no differences in age, sex, body weight, primary operative method, hernial laterality, defect size, or herniated organ between the two groups. The duration from the first operation to re-operation was 13.9 months (range: 1–96 months) in the HL group and 20.2 months (range: 0.3–80 months) in the IPTR group. All recurrent PIH cases were of indirect hernia. The surgical outcomes are shown in Table 2. There were no differences in operation time, length of hospital stay, or complications between the two groups. Re-recurrence only occurred in the HL group; no patients in the IPTR group developed re-recurrence (8.8% [5/57] vs. 0.0% [0/63], respectively). HL was a significant risk factor for re-recurrence (*p* = 0.044). The mean duration from re-operation to re-recurrence in these five patients was 10.6 months. Three patients developed re-recurrence within 1 year after re-operation, and the remaining two patients developed re-recurrence 21 and 22 months after re-operation, respectively.

**Table 1:** Patient demographics

	HL (*N* = 58)	IPTR (*N* = 68)	*p* value*
Age at re-operation (months)ª	45.4 ± 30.5 (2–118)	44.2 ± 32.1 (3–119)	0.805
Sex			0.552
Male	51 (87.9%)	63 (92.6%)	
Female	7 (12.1%)	5 (7.4%)	
Body weight (kg)ª	16.6 ± 6.9 (5.0–35.0)	18.6 ± 12.2 (4.5–83.0)	0.784
Interval from first operation to re-operation (months)	13.9 ± 16.9 (1.0–96.0)	20.2 ± 19.8 (0.3–80.0)	0.099
Operative method at first operation			0.232
Open	31 (53.5%)	26 (38.2%)	
Laparoscopy			
Extraperitoneal	18 (31.0%)	28 (41.2%)	
Intracorporeal	9 (15.5%)	14 (20.6%)	
Laterality			0.203
Right	31 (53.4%)	45 (66.2%)	
Left	27 (46.6%)	23 (33.8%)	
Herniated organs			0.781
Bowel	14 (24.1%)	13 (19.1%)	
Ovary	1 (1.7%)	1 (1.5%)	
Omentum	43 (74.2%)	54 (79.4%)	
Defect size (cm)ª (Diameter of deep inguinal ring)	1.8 ± 0.3 (1.5–2.5)	1.9 ± 0.3 (1.7–2.9)	0.109
Follow-up period (months)ª	71.1 ± 12.2 (56–95)	39.7 ± 8.8 (26–55)	< 0.001

*HL* high ligation; *IPTR* iliopubic tract repair

Values are presented as mean ± standard deviation or number (%)

^*^χ^2^ test or Fisher’s exact test

ªWilcoxon rank-sum test

**Table 2:** Surgical outcomes

	HL (*N* = 58)	IPTR (*N* = 68)	*p* value*
Operation time (min)ª	16.8 ± 5.9 (9–29)	18.5 ± 7.2 (9–35)	0.392
Postoperative hospital stay (h)ª	10.6 ± 9.0 (4–48)	8.5 ± 4.1 (5–30)	0.618
Complication	2 (3.4%)	2 (2.9%)	0.987
Hematoma	1	1	
Seroma	1	1	
Wound infection	0	0	
Intraabdominal organ injury	0	0	
Chronic inguinodynia	0	0	
Atrophic testis	0	0	
Return to normal activity (Postoperative day)ª	3.5 ± 1.1 (2–6)	3.4 ± 1.2 (2–5)	0.427
Re-recurrence, N (%)	5 (8.6%)	0 (0.0%)	0.044
Re-recurrence period (months)ª	10.6 ± 9.6 (3–22)		N/A

^*^χ^2^ test or Fisher’s exact test

ªWilcoxon rank-sum test

### Specific appearance of recurrent PIH

**Fig. 4:**
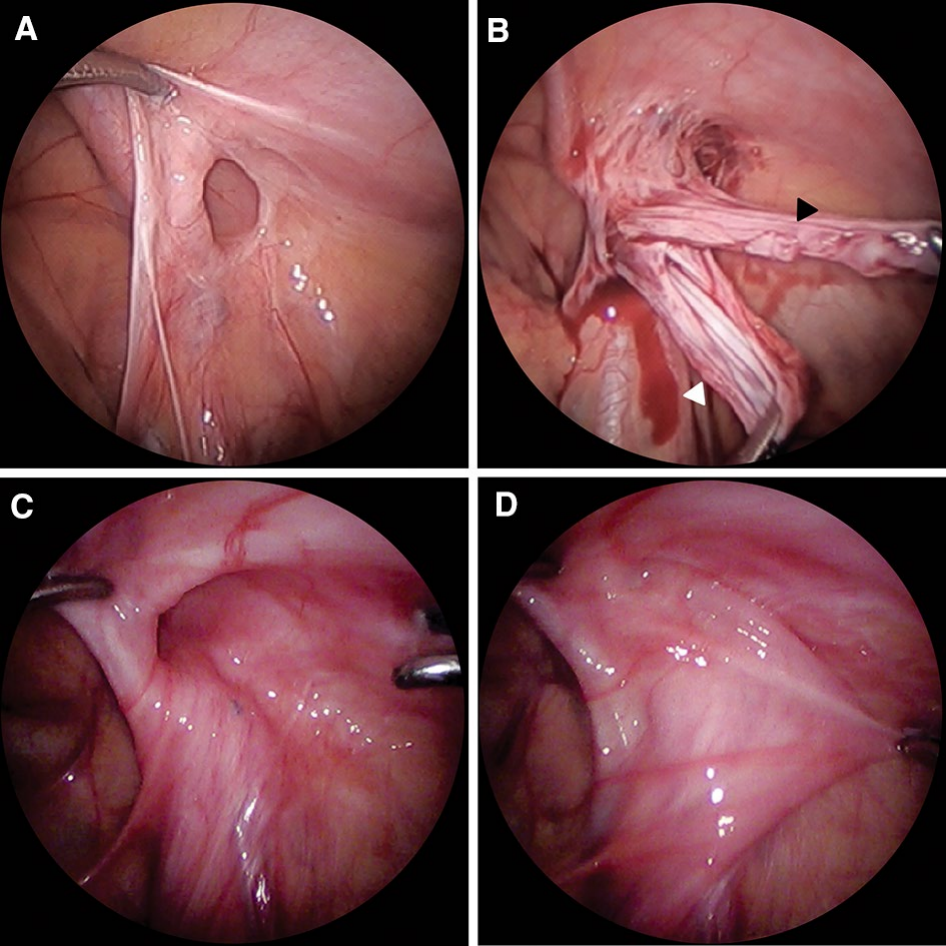
Specific appearance of recurrent PIH. **A** Recurrent PIH. **B** Two hernial sacs were observed due to formation of another peritoneal hernial sac (black arrowhead) in addition to the hernial sac (white arrowhead) that was not removed during the first operation. **C** The internal ring was widened. **D** When the hernial sac was pulled into the abdominal cavity, no loosened hernial sac was observed. *PIH* pediatric inguinal hernia

We experienced an acquired hernia defect in recurrent PIH repair. In some cases, there was an acquired hernial sac in addition to the congenital hernial sac (Fig. 4A). This shows that the congenital defect was closed in the first operation, but another hernial sac formed as an acquired hernia defect developed, which supports the notion that recurrent PIH is caused by an acquired defect. In addition, as the internal ring is widened during recurrent PIH repair, there is sometimes no loosened hernia sac observed when the hernial sac is pulled into the abdominal cavity (Fig. 4B). This situation is different from that of the typical congenital patent PV.

## Discussion

Most cases of PIH are caused by congenital defects due to open PV or CN. However, PIH can also occur due to acquired opening of the internal inguinal ring. Using the Nyhus classification system, PIH is classified as Nyhus type 1 (congenital patent PV) [17], while recurrent PIH is the caused by an acquired defect. This supports that re-operation of recurrent PIH should be different from that of primary PIH.

Laparoscopic PIH repair is performed using various methods. Many methods are used to treat the hernial sac, including complete peritoneal disconnection, but not removal; hernial sac removal; and neither peritoneal disconnection nor hernial sac removal [18]. The recurrence rate is low with sac disconnection and removal surgery according to previous studies [19, 20]. In the present study, hernial sac removal was performed in both the HL group and the IPTR group. In terms of the suture material, two materials are often used: absorbable tie and non-absorbable tie. One study indicated that use of non-absorbable suture decreases the rate of recurrence [21]. In the present study, non-absorbable suture was used in both groups. Many reports have compared open repair with laparoscopic repair for PIH treatment, and there appears to be no difference in the rate of recurrence between these approaches [22, 23].

Laparoscopic PIH repair uses different approaches for LTAC and LPEC, but these two methods cannot be compared by simple differences in their approaches, since the technical details of these procedures, including sac treatment, suture material, and suture method, are very different. The sac treatment, suture material, and suture method should be the same in order to fairly compare open repair surgery with laparoscopic repair in PIH treatment. To compare LPEC with LTAC in PIH treatment, it is necessary to compare the technical details. For example, in one paper comparing LPEC with LTAC [24], the suture material affected the rate of recurrence.

As an open approach, IPTR has been applied for the treatment of adult inguinal hernia for decades. There have been many reports of primary PIH and recurrent PIH treated using the laparoscopic approach [7, 11, 12, 25]. However, there are several concerns about IPTR. The first is injury to the vas deferens and spermatic cord when removing the hernial sac [26]. We encountered no intraoperative major bleeding or vas deferens injury due to removal of the hernial sac while separating the vas deferens and spermatic cord from the hernial sac (Fig. 3). The first stitch was sutured with a space for the vas deferens and spermatic cord to pass through (Fig. 3). The second concern is pain due to tension caused by suture. However, pain is difficult to assess in pediatric patients. In studies of laparoscopic IPTR, there was no difference in pain between patients who underwent HL and those who underwent IPTR [11, 12]. The third concern is nerve injury. The entry point of the femoral branch of the genitofemoral nerve is in the caudal location of the inguinal ligament in 84.0% of patients and in the medial direction in the anterior superior iliac spine in 5.2% of patients [27]. Therefore, when suturing the iliopubic tract, sutures should be placed as close as possible to the inguinal ring. The fourth concern is technical difficulty. One report suggested no difference in operation time between HL and IPTR in laparoscopic repair of primary PIH [11]. In this study, which used laparoscopic repair for recurrent PIH, there was no difference in operation time between HL and IPTR. At the beginning of this study, laparoscopic IPTR took 25–30 min, but over 100 cases, the operation took 10–15 min. Thus, laparoscopic IPTR poses a learning curve, but it is not difficult.

The suture approach is comprised of three stages in laparoscopic re-operation for recurrent PIH: simple HL without disconnection of the distal hernial sac, transection and removal of the hernial sac (the advanced stage), and removal of the hernial sac and IPTR (the most advanced stage). In some reports, the rate of recurrence after laparoscopic IPTR for the treatment of primary PIH was low [11, 25]. In the present study, HL was a significant risk factor for re-recurrence after laparoscopic repair of recurrent PIH. Five patients in the HL group developed re-recurrence, while no patients in the IPTR group developed re-recurrence. Postoperative complications did not differ between groups in this study.

This study has several limitations, including its retrospective design. The follow-up period differed between the two groups in this study. However, all five cases of re-recurrence developed in the HL group within 22 months, and 26 months have passed since the last patient was treated in the IPTR group. Thus, the effect of this difference in the follow-up period is expected to be minimal. Because the follow-up period was short, longer follow-up is required to ascertain the actual re-recurrence rate. All operations were performed by a single surgeon, and there were no changes in operative facilities during the study period. Therefore, IPTR may be considered an effective operation that does not increase the complication rate and that reduces re-recurrence in the treatment of recurrent PIH. Because this study was a single-center study, further multi-center studies are needed in the future.

In conclusion, IPTR significantly reduces the re-recurrence rate of recurrent PIH compared with HL. Therefore, laparoscopic IPTR is an effective and safe laparoscopic treatment for recurrent PIH.
